# Prevention of Maternal–Fetal Transmission of Cytomegalovirus

**DOI:** 10.1016/j.ebiom.2015.08.004

**Published:** 2015-08-05

**Authors:** Stuart P. Adler

**Affiliations:** CMV Research Foundation Inc., Richmond, VA 23298, United States

Annually in the United States approximately 40,000 pregnant women are infected with CMV (seroconvert) during pregnancy and probably an equal number in Europe. Of those seroconverting during pregnancy approximately 20% of their infants develop neurologic damage and/or hearing deficit. A hopefully final and definitive report by Revello et al. in this journal confirms that the majority of these infections are easily prevented by simple hygienic precautions (Revello et al., 2015).

Between 20% and 60% of women are susceptible (seronegative) to CMV at conception. Maternal immunity from a pre-conception CMV infection protects against a second CMV infection and protects the fetuses from severe postnatal neurosensory deafness and neurologic damage. Thus a primary maternal infection with CMV in early pregnancy causes the majority of congenital disease. After a primary infection during pregnancy, the fetal infection rate varies from 33% to 75% as gestation progresses and disease rates may be as high as 50% if infection occurs during early gestation (Bodéus et al., 2010; Nigro et al., 2005).

The majority of seronegative pregnant women acquire CMV from a child less than three years of age in the home or for pregnant women employed in infant day care centers, from children in their care (Adler et al., 2004; Adler, 1989). CMV seronegative health care providers caring for hospitalized young children and infants, are not at an increased risk (Adler, 2010). Infants acquire CMV in utero, via breast milk, or via contact with other children. These CMV infected infants, unlike older children and adults, have CMV in urine and saliva for an average of 18 months (Adler, 1991). In the US, 60% of the mothers of children in daycare are CMV seronegative, and at least 25% of all young children attending large group child care centers are shedding CMV. The annual infection rate for seronegative women without exposure to children is 2%, but 5 to 25 times higher for exposed women (Adler et al., 2004; Adler, 1989).

The U.S. Centers for Disease Control suggests that pregnant women reduce their risk of CMV acquisition during pregnancy using simple hygienic precautions but this suggestion is not often followed. Studies demonstrating the efficacy of hygienic precautions are compelling (Revello et al., 2015; Adler et al., 1996, 2004; Finney et al., 1993; Vauloup-Fellous et al., 2009). Our U.S. studies of CMV seronegative pregnant women with an infected young child found that the precautions were highly effective (p < 0.008) (Adler et al., 1996, 2005, 2015). Women were told about CMV, provided written guidelines detailing hygienic precautions, and provided a demonstration video. None declined testing and none complained the precautions were difficult or provoked anxiety. Overall of 37 pregnant women with a child shedding CMV, only one (3%) who received hygienic precautions seroconverted to CMV during pregnancy. This contrasts with an infection rate of 42% for 154 non-pregnant women and women trying to conceive who also had infected children.

A French study offered 5312 pregnant women CMV serologic screening at 12 week gestation and 97.4% agreed (Vauloup-Fellous et al., 2009). Seronegative women and their spouses received oral and written hygienic precautions. For 2595 seronegative women, the rate of maternal seroconversion during the first 12 weeks of gestation was compared to the rate between weeks 12 and 36. Prior to the receipt of hygienic precautions at 12 weeks the maternal seroconversion rate when adjusted for the number of woman–weeks observed was 0.035% per woman–week compared to a rate of 0.008% per woman–week after intervention (P = 0.0005). Maternal primary infections and seroconversions were distributed evenly throughout gestation.

Revello et al. in Italy prospectively observed that of 331 CMV seronegative pregnant women who receive oral and written hygienic precautions for preventing CMV acquisition from a young child only four (1.2%) women seroconverted to CMV during pregnancy (Revello et al., 2015). In contrast of 315 women in a comparison group, CMV infection occurred in 24 (7.6%) women during pregnancy.

In each report, the efficacy of hygienic precautions has been > 75%. Given the ease and effectiveness of this intervention it is appropriate that CMV education and hygienic precautions be implemented clinically. All pregnant women with frequent contact with a child < 3 years of age should be identified as early in gestation as possible and offered serologic testing (IgG antibodies to CMV only). If seronegative, women should be given the precautions. Subsequent testing during pregnancy would be optional based on the level of concern of the woman and the results of ultrasound findings. Screening for fetal disease by ultrasound is nearly universal in developed countries. If ultrasound findings are normal, the risk of CMV fetal or neonatal disease is low and hence repeat testing should be unnecessary (Nigro et al., 2005).

In each published study women knew they were seronegative and hence at risk. Is serologic testing necessary for motivation and for the precautions to be effective? We do not know. Also we do not know how often seropositive women become reinfected during pregnancy and if maternal reinfection leads to fetal disease. These questions can be answered in subsequent studies but are not reasons to delay implementation of the precautions for seronegative women.

Serologic screening of high risk pregnant women for CMV is as feasible as is current screening for syphilis, hepatitis, rubella, and HIV. The assays for IgG to CMV have an accuracy equal or great than the accuracy for the other infections. Serologic screening for CMV IgM or IgG avidity should not be done.

Although seronegative health care workers are not at risk, pregnant childcare employees are. Pregnant childcare employees should be informed about CMV, assess their risk by serologic testing or avoid if possible caring for children less than 2 years age for the duration of pregnancy. The efficacy of hygienic precautions for childcare employees is unknown.

For seronegative pregnant women who are at high risk because of exposure to a young child in the home or in large group childcare, hygienic precautions are simple, inexpensive, and highly effective.

## Conflict of Interests

None.
